# More is not always better: phosphorus context shapes the effectiveness of single-strain and consortium plant growth-promoting bacteria in tomato

**DOI:** 10.1007/s00425-026-05134-4

**Published:** 2026-08-25

**Authors:** Alice Ferreira Alves, Mónica Yorlady Alzate Zuluaga, Talita de Oliveira Caretta, Sonia Monterisi, Fernando Dini Andreote, Stefano Cesco, Youry Pii

**Affiliations:** 1 Luiz de Queiroz College of Agriculture (ESALQ), University of São Paulo, Piracicaba, São Paulo 13418-900 Brazil; 2 Faculty of Agricultural, Environmental and Food Sciences, Free University of Bolzano, Piazza Università 5, 39100 Bolzano, Italy

**Keywords:** Bacterial inoculant, Multi-strain formulation, Nutrient network, P availability

## Abstract

**Main conclusion:**

**Phosphorus availability determines whether single or consortium PGPB maximize tomato performance, demonstrating that rational inoculant design should prioritize functional complementarity over increasing microbial complexity.**

**Abstract:**

Phosphorus (P) availability is a major constraint for crop development and productivity, and beneficial bacteria are increasingly proposed to enhance plant P-acquisition efficiency. However, their comparative performance remains variable across studies and experimental contexts. This study investigated whether single plant growth-promoting bacteria (PGPB) strains and a defined bacterial consortium differentially modulate P nutrition and early growth of tomato under contrasting P availability. Tomato plants were grown hydroponically in a factorial design combining single-strain inoculations (*Azospirillum brasilense*, *Pantoea agglomerans*, *Priestia aryabhattai*), a defined consortium, and a non-inoculated control under five P regimes differing in concentration and chemical form (soluble *vs.* insoluble). Plant growth and root architecture traits were evaluated alongside ionomics and targeted expression analysis of *SlPHT1* transporters, thus linking plant performance with nutrient status and regulation. Results indicated that *P. agglomerans* showed the most stable benefits under soluble P, while *A. brasilense* strongly promoted adaptive root and transporter responses under moderate limitation. *P. aryabhattai* induced subtler growth effects but reshaped nutrient associations, particularly with insoluble P. By contrast, the consortium did not consistently outperform single strains and showed clear advantages only when insoluble P was the sole source. Overall, these findings demonstrate that the effectiveness of single *versus* multi-strain inoculation is strongly context-dependent and emerges from the interaction between P availability, strain identity, and plant response integration. Accordingly, effective consortia should be designed based on functional complementarity and balance, rather than on increasing inoculant complexity, as overlapping PGP traits may lead to non-additive or even counterproductive effects.

**Supplementary Information:**

The online version contains supplementary material available at https://doi.org/10.1007/s00425-026-05134-4.

## Introduction

Phosphorus (P) plays a central role in plant growth and crop productivity, as it is required for key physiological and developmental processes, including root development, blooming, seed formation, photosynthesis, and maturation (Rawat et al. 2021). However, its limited availability in soils often poses a significant stress for plants, constraining agricultural productivity and making P one of the most limiting nutrients in farming systems worldwide. This limited availability largely arises from the strong affinity of P for soil minerals, which causes it to bind tightly to particles and become unavailable for plant uptake. In mineral soils, inorganic soluble P readily forms poorly soluble complexes with iron (Fe), aluminum (Al), and calcium (Ca), depending on soil pH and geochemical conditions (Castagno et al. 2021). In addition, a substantial proportion of soil P occurs in organic forms that require biological mineralization before becoming available for plant uptake and may also be stabilized through associations with metal cations. As a consequence, only a small fraction of applied P fertilizers is recovered by crops, with most of the input rapidly immobilized in the soil matrix or lost through transport processes (Ogwu et al. 2025). This low recovery efficiency not only represents a major economic cost but also exacerbates environmental pressures, including nutrient accumulation in soils and eutrophication of adjacent aquatic ecosystems. Therefore, improving plant P acquisition and use efficiency remains a major challenge for the transition toward more sustainable agricultural systems, and, more broadly, for the development of farming systems in which belowground processes contribute not only to stress mitigation but also to a more antifragile functional organization under fluctuating environmental conditions (Cesco et al. 2026).

In light of these challenges, increasing attention has been directed toward biological strategies capable of enhancing P-acquisition efficiency. Among these, the application of plant growth-promoting bacteria (PGPB) has emerged as a promising alternative for supporting crop nutrition while reducing dependence on mineral fertilizers (Shayanthan et al. 2022). These beneficial microorganisms can establish themselves in the rhizosphere or colonize internal plant tissues, where they interact closely with roots and influence plant performance through a range of complementary mechanisms (Al Raish et al. 2025). Over the past 2 decades, numerous strains with plant growth-promoting traits have been isolated, characterized, and applied as inoculants to enhance crop productivity (Bisht et al. 2024). These functions include, among others, P solubilization and modulation of root traits involved in nutrient acquisition, thereby affecting plant nutritional status in a context-dependent manner. Beyond their nutritional roles, PGPB can also enhance plant tolerance to environmental constraints by contributing to defense responses against pathogens and by mitigating the effects of abiotic stresses such as drought and salinity, ultimately fostering healthier and more sustainable crop development (Zuluaga et al. 2021).

Currently, an important development in the application of PGPB is the use of defined bacterial consortia, in which multiple functionally complementary strains are combined into a single inoculant. These consortia are assembled through the targeted selection of bacterial species with distinct yet complementary traits, with the aim of enhancing nutrient acquisition, plant productivity, and resilience to biotic and abiotic stresses by simultaneously activating multiple growth-promoting mechanisms (Santoyo et al. 2021). Compared with single-strain inoculants, bacterial consortia can provide a broader functional spectrum, including improved nutrient mobilization, modulation of root system development, and enhanced root–microbe contact, which collectively support plant growth and nutritional status (Bradáčová et al. 2019). Accordingly, consortia are increasingly regarded as promising biobased tools for improving crop performance under nutritionally constraining conditions, although their effectiveness is not always consistent.

The effectiveness of bacterial consortia is strongly influenced by the availability of resources in the rhizosphere and by the degree of functional complementarity among consortium members. Combining strains with different metabolic capacities can reduce direct competition for substrates, promote niche differentiation, and improve the stability of microbial functions related to nutrient cycling (Benmrid et al. 2025). From an experimental perspective, defined consortia also offer a useful framework to investigate microbial coexistence and functional integration, allowing researchers to assess how multiple plant growth-promoting traits interact at the root–soil interface (Khan 2022). These insights are increasingly used to guide the rational selection and assembly of microbial inoculants aimed at improving nutrient use efficiency in agricultural systems. At the same time, however, the potential advantage of consortia cannot be assumed a priori, because their performance depends on whether strain interactions are truly complementary under the environmental conditions in which they are applied.

Beyond conceptual considerations, a growing body of experimental evidence indicates that bacterial consortia can enhance plant growth primarily through improvements in mineral nutrition. Several studies have reported increased biomass accumulation and enhanced uptake of essential nutrients following inoculation with multi-strain formulations, effects that are often attributed to the combined action of complementary bacterial traits rather than to a single dominant mechanism (Liu et al. 2023; Abou Jaoudé et al. 2025; Kurt 2025). However, this apparent advantage of consortia is not universal. Increasing evidence shows that individual PGPB strains can, in some cases, outperform multi-strain formulations for specific plant traits, depending on the functional roles of the strains involved and their interactions within the consortium. For instance, Aleksieienko et al. (2025) showed that *Pseudomonas* sp. promoted *Arabidopsis* seedling development more effectively than several consortia. Such outcomes indicate that the presence of multiple strains does not necessarily lead to additive or synergistic responses, as competition, functional redundancy, or antagonism within consortia may limit overall performance (Timofeeva et al. 2023). This issue is particularly relevant in the case of P nutrition, where the effectiveness of microbial inoculation is expected to depend not only on the functional traits of the strains, but also on P form, P availability, and plant developmental stage.

Overall, these contrasting observations suggest that the relative effectiveness of single-strain versus consortium-based inoculation depends on the specific experimental conditions and remains unclear. Despite the increasing literature on microbial consortia, systematic comparisons between defined consortia and their individual constituent strains are still limited, particularly under controlled nutrient regimes and during early stages of plant development, when plant establishment is strongly influenced by nutrient acquisition efficiency and root-microbe interactions. Addressing this gap requires experimental systems that minimize environmental variability and allow microbial and nutritional effects to be evaluated separately. This need is especially relevant in tomato (*Solanum lycopersicum* L.), a crop of agronomic importance and a widely used model for investigating nutrient uptake responses under controlled conditions. In this context, the present study evaluated the performance of a defined bacterial consortium relative to its individual PGPB components in tomato plants, focusing on P nutrition and early growth under controlled hydroponic conditions. The experimental design integrated graded P levels supplied as soluble and insoluble inorganic sources, alongside morpho-physiological, ionomic, and transcriptional analyses. This multilevel approach aimed to determine not only whether single strains and the consortium differ in effectiveness, but also how these differences manifest across plant functional organization under contrasting P regimes and forms.

## Materials and methods

### Screening of bacterial strains

A total of fifteen bacterial isolates from the culture collection of the Plant and Microbe Biotechnology research group at the Free University of Bolzano (Italy) were screened for functional traits associated with plant growth promotion. The screening focused on three key functions: phosphate solubilization, nitrogen fixation, and exopolysaccharide (EPS) production. Phosphate-solubilizing activity was assessed on solid medium supplemented with insoluble phosphate, where the formation of clear halos around bacterial colonies was used as an indicator of solubilization capacity, following the method of Nautiyal (1999). Nitrogen fixation ability was assessed using the N-free JNFb medium, following the method described by Baldani et al. (1997). EPS production, used as an indicator of biofilm-forming capacity, was quantified in liquid culture using a colorimetric assay based on the phenol–sulfuric acid method of DuBois et al. (1956). Based on performance across these assays, three strains were selected, each exhibiting the highest activity within one functional category: *Azospirillum brasilense* (N fixation), *Pantoea agglomerans* (P solubilization), and *Priestia aryabhattai* (EPS production). To verify their compatibility, the selected strains were subjected to cross-streaking on LB agar plates. The absence of visible antagonistic interactions indicated that the strains were compatible and suitable for use in subsequent co-inoculation experiments (Schwyn and Neilands 1987).

### Bacterial treatments

Each bacterial strain was grown separately in LB broth (10 g L⁻1 tryptone, 5 g L⁻1 yeast extract, 10 g L⁻1 NaCl) at 28 °C with constant shaking at 180 rpm, for 24 h. After incubation, cells were harvested, washed, and resuspended in sterile saline solution (0.85% w/v NaCl). Cell density was quantified with a Neubauer counting chamber, and suspensions were adjusted to 10⁶ cells mL⁻1. For the bacterial consortium, equal volumes of the standardized single-strain suspensions were mixed. The inocula, either individual strains or consortium, were then added to the hydroponic nutrient solution (NS) to inoculate plant roots according to the respective treatments.

### Experimental design and plant growth conditions

**Fig. 1:**
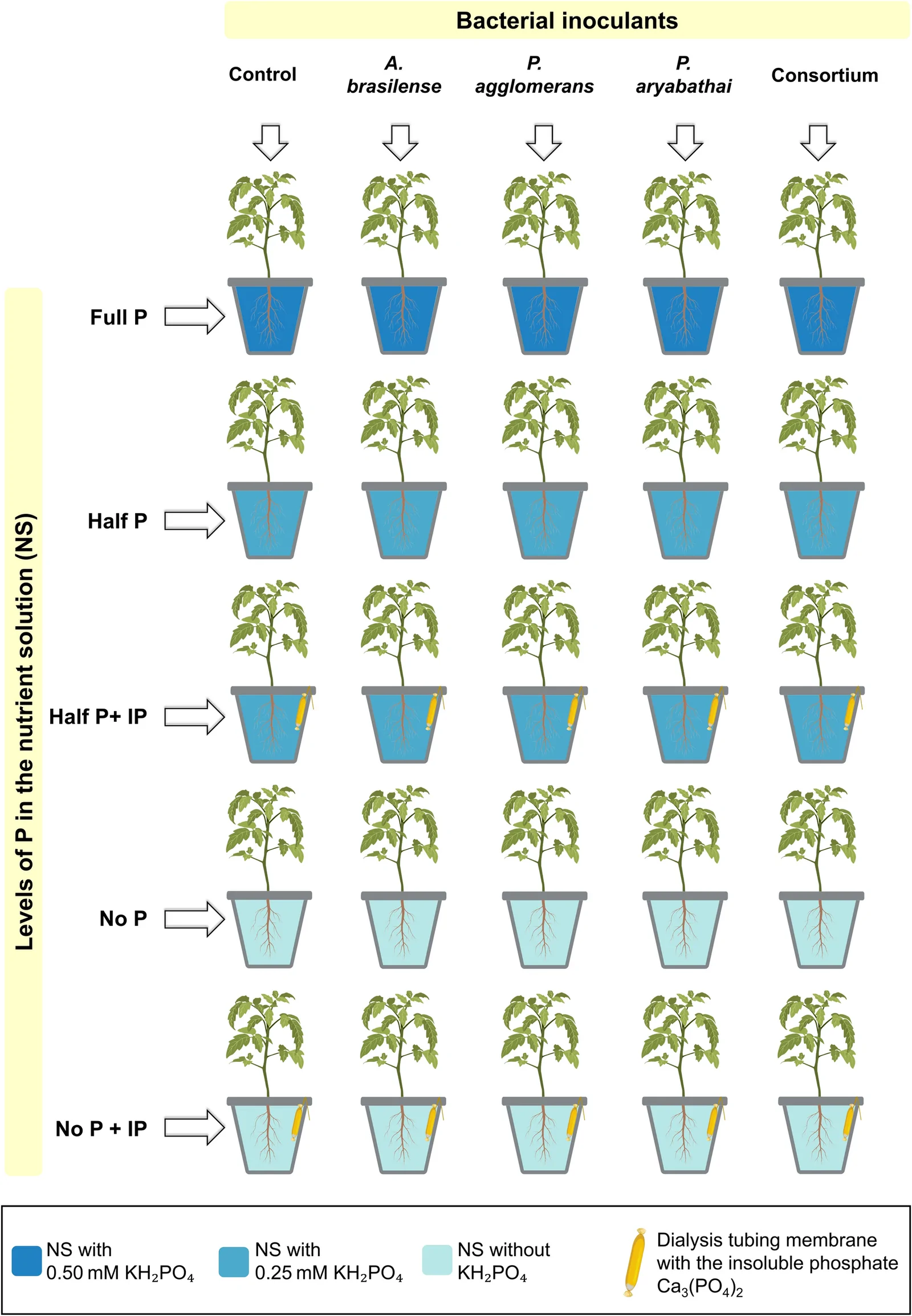
The representation of treatments applied to the nutrient solution (NS). The experiment was conducted in a 5 × 5 factorial design, combining five bacterial inoculation treatments (a non-inoculated control, *A. brasilense*, *P. agglomerans*, *P. aryabhattai*, and the consortium) with five phosphorus levels: Full P (complete concentration), Half P (50% of the recommended dose), Half P + IP (50% dose with addition of an insoluble phosphorus source), No P (no phosphorus), and No P + IP (no phosphorus with addition of an insoluble source). The dialysis tubes used were a MEMBRA-CEL^®^ MD34 membrane (14 × 100) with a molecular weight cut-off of 14,000 Da and a pore size of 50 Å (Viskase Companies Inc., Willowbrook, IL, USA)

Tomato (*Solanum lycopersicum* L.) seeds were germinated for 5 days in the dark at 22 °C on filter paper moistened with distilled water. After the germination period, the seedlings were transplanted into pots containing 1.5 L of a modified Hoagland nutrient solution (NS) composed of 1 mM MgSO₄, 2 mM Ca(NO₃)₂, 0.1 mM KCl, 1 mM K₂SO₄, 10 μM H₃BO₃, 0.5 μM MnSO₄, 0.2 μM CuSO₄, 0.5 μM ZnSO₄, 0.01 μM (NH₄)₆Mo₇O₂₄, and 80 μM FeEDTA. The experiment was established as a 5 × 5 factorial in a completely randomized design (Fig. 1), combining five bacterial inoculation treatments (control, *A. brasilense*, *P. agglomerans*, *P. aryabhattai*, and the consortium) with five P regimes levels (Full P, Half P, Half P+IP, No P, and No P+IP). Phosphorus treatments were applied from the beginning by varying the concentration of soluble P supplied as KH₂PO₄ in the NS and, when applicable, supplementing with insoluble Ca₃(PO₄)₂ placed inside dialysis tubes. Specifically, Full P consisted of 0.5 mM KH₂PO₄, Half P contained 0.25 mM KH₂PO₄, Half P+IP included 0.25 mM KH₂PO₄ plus 50% of the total P as Ca₃(PO₄)₂, No P excluded KH₂PO₄ entirely, and No P+IP provided P solely as Ca₃(PO₄)₂ (100% insoluble P). To maintain stable growth conditions, the NS was adjusted to pH 6.0 with KOH and fully renewed twice per week to avoid nutrient depletion and pH drift. Dialysis tubes containing Ca₃(PO₄)₂ were also replaced at each NS renewal to maintain consistent P availability across the experimental period. Bacterial inoculation was performed once at the beginning of the experiment by adding suspensions directly into the NS at the root zone to a final density of 10⁶ cells mL⁻^1^. Although root colonization was not directly verified, the strategy was designed to promote early association between bacteria and roots, and no re-inoculation was performed during subsequent NS replacements. The NS was continuously aerated and renewed twice a week. Each treatment consisted of three biological replicates, with ten seedlings per replicate. After 21 days of cultivation, plants were harvested and evaluated as described below.

### Plant growth and elemental analysis

After 21 days of hydroponic cultivation, plants were harvested for growth and physiological assessments. Chlorophyll concentration was determined on the youngest fully expanded leaves using a portable chlorophyll meter (SPAD-502, Minolta) and expressed as soil–plant analysis development (SPAD) units. Measurements were performed on three plants per biological replicate, with at least two readings per plant, and the average value was used to represent each replicate. For destructive analyses, one plant from each biological replicate (*n*=3) was used for root morphology, leaf area, biomass, and elemental analyses. Fresh root systems were scanned using a root scanner (EPSON Perfection V800, Regent Instruments Inc.), and root traits, including total length, surface area, and volume, were quantified using WinRHIZO software (WinRHIZO Pro 2003b, Regent Instruments Inc.). Total leaf area was estimated from digital photographs using Easy Leaf Area software (Easlon and Bloom 2014). The same plants were subsequently dried at 65 °C until constant weight for determination of shoot and root dry weight (DW) and were then used for mineral nutrient analysis. Dried shoot and root samples were acid-digested using 68% ultrapure HNO₃ (Carlo Erba, Cornaredo, Milan, Italy) in a single-reaction-chamber microwave digestion system (UltraWAVE, Milestone, Sorisole, Italy). Concentrations of macro- and micronutrients were determined by inductively coupled plasma-optical emission spectrometry (ICP-OES; Spectro Ciros CCD, Spectro, Kleve, Germany). Element quantification was performed using certified multi-element standards (CPI International, Santa Rosa, CA, USA). Tomato leaves (SRM 1573a) and spinach leaves (SRM 1547) were used as external certified reference materials.

### Gene expression analysis

Root tissues from two plants within each biological replicate were pooled to obtain one biological sample (*n* = 3), immediately frozen in liquid nitrogen, and stored at −80 °C until further processing. Total RNA was extracted from frozen roots using the Spectrum Plant Total RNA Kit (Sigma-Aldrich) according to the instructions of the manufacturer. RNA concentration and purity were assessed spectrophotometrically, and 1 μg of total RNA was treated with 10 U of RQ1 DNase (Promega) to remove residual genomic DNA. First-strand cDNA synthesis was performed using the ImProm-II™ Reverse Transcription System (Promega) with oligo(dT)₁₅ primers, according to the manufacturer’s recommendations. The resulting cDNA was used as a template for quantitative real-time reverse transcription PCR (qRT-PCR), which was carried out using SsoFast^™^ EvaGreen^®^ Supermix (Bio-Rad) on a Bio-Rad iCycler MyiQ real-time PCR system. Gene-specific primers were designed for the target genes as well as for the reference gene elongation factor 1α (EF1α) (Suppl. Table S1). Each reaction was performed in triplicate under the following thermal cycling conditions: an initial denaturation at 95 °C for 5 min, followed by 40 cycles of denaturation at 95 °C for 30 s and annealing/extension at 55 °C for 30 s, as previously described (Pii et al. 2019). Amplification efficiencies were calculated from raw fluorescence data using LinRegPCR software Ramakers et al. (2003). For each transcript, mean normalized expression (MNE; Simon 2003) values were calculated using EF1α as the reference gene. Relative expression levels were then determined using the 2⁻^ΔΔCt^ method, as described by Livak and Schmittgen (2001).

### Statistical analysis

Results are reported as mean values based on three biological replicates, with variability expressed as the standard error (SE). The pot was considered the biological and experimental unit for all statistical analyses (*n*=3). Statistical analyses were performed using the R statistical environment (version 4.1.2). The effects of bacterial inoculation, phosphorus regime, and their interaction were evaluated using two-way analysis of variance (ANOVA). When the interaction between P level and bacterial inoculation was significant, treatment combinations were compared as independent groups using the Scott–Knott clustering procedure at a significance threshold of *P* < 0.05. This method groups treatments into statistically homogeneous classes, allowing clear identification of treatment response patterns while avoiding extensive pairwise comparisons. To investigate nutrient co-regulation patterns, network analyses using Pearson correlation coefficients were calculated among all pairs of nutrients. Edges were defined by strong correlations with an absolute coefficient |r|≥ 0.7. Correlation networks were constructed as undirected graphs, where nodes represent individual nutrients and edges represent significant associations between them. Network density (ND) was calculated as the ratio between the number of observed edges and the total possible number of edges. Mean degree was defined as twice the number of edges divided by the number of nodes. The proportions of positive and negative edges were calculated relative to the total number of significant correlations within each network. To explore overall treatment effects on plant growth traits and elemental composition, a heatmap was constructed. Prior to visualization, all variables were normalized by z score transformation, with values centered to the mean and scaled to unit variance to ensure comparability across parameters. Hierarchical clustering was then applied to the standardized dataset using Ward’s minimum variance method (ward.D2) in combination with Euclidean distance. Data processing, statistical analyses, and graphical outputs were performed using the R packages *ggplot2*, *agricolae*, *ggpubr*, *igraph,* and *pheatmap*.

## Results

### Effect of P availability and bacterial inoculation on plant morpho-physiological parameters

Two-way ANOVA revealed highly significant effects (*P* ≤ 0.001) of phosphorus level (P), bacterial inoculation (BI), and their interaction (P × BI) on all tomato growth parameters evaluated, including biomass accumulation, root morphology, leaf area, and chlorophyll index (SPAD) (Table 1). Across P levels, bacterial inoculation markedly influenced plant biomass production.

**Table 1:** Effects of bacterial inoculant treatments and P levels on tomato growth parameters

Main factors	RDW (mg)	SDW (mg)	PDW (mg)	Root length (cm)	Root surface area (cm^2^)	Root volume (cm^3^)	Total leaf area (cm^2^)	SPAD
Full P x Control	2.6 ± 0.3 e	52.0 ± 0.4 f	54.5 ± 0.3 e	140.0 ± 1.8 g	86.0 ± 0.8 h	0.043 ± 0.002 f	19.1 ± 0.5 h	26.8 ± 0.9 g
Full P x *A. brasilense*	2.1 ± 0.1 f	54.2 ± 1.3 e	56.3 ± 1.3 e	87.0 ± 2.8 j	55.4 ± 0.3 l	0.026 ± 0.001 h	19.5 ± 1.4 h	24.9 ± 0.5 h
Full P x *P. agglomerans*	6.5 ± 0.4 a	75.3 ± 1.9 b	81.7 ± 1.5 b	214.7 ± 3.2 b	114.8 ± 1.6 d	0.055 ± 0.001 d	28.8 ± 1.0 d	26.8 ± 0.2 g
Full P x *P. aryabhattai*	2.6 ± 0.2 e	45.7 ± 4.2 g	48.3 ± 4.0 f	115.1 ± 1.8 i	76.9 ± 0.3 j	0.037 ± 0.004 g	22.4 ± 0.1 g	25.2 ± 0.1 h
Full P x Consortium	4.0 ± 0.1 c	93.7 ± 1.9 a	97.7 ± 1.8 a	186.5 ± 2.9 d	113.9 ± 4.8 d	0.049 ± 0.003 e	29.9 ± 0.6 c	24.6 ± 0.6 h
Half P x Control	1.9 ± 0.3 f	43.8 ± 0.9 g	45.7 ± 0.6 f	128.5 ± 2.5 h	83.0 ± 3.0 i	0.043 ± 0.002 f	19.1 ± 0.2 h	27.5 ± 0.4 g
Half P x *A. brasilense*	4.3 ± 0.5 c	75.0 ± 2.0 b	79.3 ± 1.8 b	111.5 ± 4.5 i	78.0 ± 2.0 j	0.036 ± 0.006 g	23.8 ± 0.2 f	26.1 ± 0.7 g
Half P x *P. agglomerans*	5.0 ± 0.7 c	65.2 ± 1.1 c	70.2 ± 1.8 d	256.5 ± 17.5 a	118.3 ± 1.2 c	0.054 ± 0.001 d	43.7 ± 1.0 a	19.6 ± 0.4 j
Half P x *P. aryabhattai*	3.6 ± 0.1 d	55.4 ± 0.8 e	59.0 ± 0.8 e	142.0 ± 5.0 g	86.0 ± 3.0 h	0.042 ± 0.004 f	31.8 ± 0.8 b	24.3 ± 1.2 i
Half P x Consortium	1.6 ± 0.1 f	55.9 ± 0.6 e	57.5 ± 0.5 e	108.7 ± 0.6 i	67.7 ± 0.6 k	0.034 ± 0.001 g	26.6 ± 0.2 e	24.9 ± 0.4 h
Half P+IP x Control	2.4 ± 0.4 e	36.6 ± 2.5 h	39.0 ± 2.7 g	133.5 ± 2.4 h	83.8 ± 2.0 i	0.050 ± 0.003 e	18.0 ± 0.8 i	20.2 ± 0.2 j
Half P+IP x *A. brasilense*	5.7 ± 0.4 b	67.3 ± 2.2 c	73.0 ± 2.4 c	166.6 ± 1.1 e	105.2 ± 1.5 e	0.053 ± 0.001 d	28.7 ± 1.3 d	24.1 ± 0.4 i
Half P+IP x *P. agglomerans*	3.8 ± 0.4 d	75.7 ± 2.9 b	79.5 ± 3.3 b	154.2 ± 3.8 f	96.7 ± 1.4 f	0.050 ± 0.001 e	31.5 ± 1.1 b	25.0 ± 0.1 h
Half P+IP x *P. aryabhattai*	4.5 ± 0.4 c	54.5 ± 3.9 e	59.0 ± 4.3 e	130.7 ± 1.9 h	83.0 ± 1.7 i	0.046 ± 0.003 f	30.2 ± 0.5 c	23.1 ± 0.6 i
Half P+IP x Consortium	2.4 ± 0.4 e	24.7 ± 0.6 i	27.1 ± 0.9 i	115.1 ± 0.4 i	77.9 ± 0.7 j	0.042 ± 0.001 f	23.6 ± 0.0 f	24.6 ± 0.1 h
No P x Control	4.3 ± 0.1 c	26.9 ± 2.8 i	31.2 ± 2.9 h	132.0 ± 5.0 h	106.3 ± 4.3 e	0.065 ± 0.003 c	4.3 ± 0.1 n	37.0 ± 0.1 a
No P x *A. brasilense*	6.9 ± 0.4 a	43.2 ± 1.7 g	50.1 ± 1.3 f	206.8 ± 4.3 c	140.5 ± 6.5 a	0.085 ± 0.004 a	9.9 ± 0.0 l	33.1 ± 0.8 c
No P x *P. agglomerans*	6.5 ± 0.5 a	51.5 ± 1.3 f	58.0 ± 1.1 e	162.0 ± 0.0 e	106.0 ± 1.0 e	0.055 ± 0.001 d	11.1 ± 0.9 k	20.8 ± 1.2 j
No P x *P. aryabhattai*	3.2 ± 0.2 d	34.5 ± 1.2 h	37.7 ± 1.3 g	168.0 ± 3.0 e	112.5 ± 0.5 d	0.060 ± 0.001 c	7.7 ± 0.2 m	34.8 ± 0.8 b
No P x Consortium	4.6 ± 0.3 c	45.0 ± 1.7 g	49.6 ± 1.5 f	148.5 ± 1.5 f	90.8 ± 1.8 g	0.054 ± 0.007 d	8.4 ± 0.3 m	28.8 ± 0.4 f
No P+IP x Control	4.4 ± 0.4 c	21.7 ± 1.6 j	26.1 ± 1.2 i	138.4 ± 2.0 g	114.0 ± 2.5 d	0.051 ± 0.003 e	4.4 ± 0.4 n	32.3 ± 0.4 d
No P+IP x *A. brasilense*	4.5 ± 0.3 c	44.5 ± 1.1 g	49.0 ± 1.2 f	164.1 ± 4.0 e	125.5 ± 0.1 b	0.075 ± 0.004 b	9.3 ± 0.3 l	33.8 ± 0.7 c
No P+IP x *P. agglomerans*	4.7 ± 0.3 f	37.4 ± 1.8 h	39.0 ± 1.5 g	201.7 ± 0.3 c	126.7 ± 0.3 b	0.063 ± 0.001 c	13.0 ± 0.3 j	24.0 ± 0.4 i
No P+IP x *P. aryabhattai*	6.0 ± 0.2 b	41.4 ± 0.5 g	47.4 ± 0.7 f	187.3 ± 5.7 d	109.8 ± 1.4 e	0.053 ± 0.001 d	11.9 ± 0.6 k	35.3 ± 1.6 b
No P+IP x Consortium	5.9 ± 0.1 b	62.0 ± 0.5 d	67.9 ± 0.6 d	171.7 ± 9.1 e	75.2 ± 4.2 j	0.057 ± 0.005 d	8.6 ± 0.2 m	31.0 ± 0.9 e
Significance								
P level (*P*)	***	***	***	***	***	***	***	***
Bacterial inoculant (BI)	***	***	***	***	***	***	***	***
P x BI	***	***	***	***	***	***	***	***

All data are expressed as mean ± SE, *n* = 3

*RDW* root dry weight, *SDW* shoot dry weight, *PDW* plant dry weight

Two-way ANOVA followed by Scott–Knott test (*P*=0.05) was applied for each growth parameter

Different letters within each column indicate significant differences according to Scott–Knott test

^***^ denotes significance at *P* ≤ 0.001

Under Full P conditions, inoculation with *P. agglomerans* and the bacterial consortium significantly increased root dry weight (RDW) by 150% and 84%, respectively, relative to the uninoculated control. Both treatments also enhanced shoot dry weight (SDW) and plant dry weight (PDW), with increases of about 45% and 80%, respectively. Notably, *P. agglomerans* induced the strongest stimulation of root biomass and morphological traits, whereas the consortium effect was more pronounced on shoot growth.

At reduced P supply (Half P), *P. agglomerans* exerted a particularly strong effect on root system development, resulting in significantly greater root length (99%), surface area (42%), and volume (26%) relative to the control and all other inoculation treatments (Table 1). In contrast, biomass responses under this P regime varied among strains. Single-strain inoculations generally performed better than the consortium, with *A. brasilense* promoting higher shoot and total plant dry weight, whereas the consortium failed to induce consistent improvements in biomass accumulation compared with individual strains.

When soluble and insoluble P were supplied together (Half P + IP), *A. brasilense* and *P. agglomerans* exhibited the strongest positive effects on tomato growth. Both strains increased shoot and total plant dry weight by more than 80% relative to the uninoculated control. In addition, *A. brasilense* markedly enhanced root system development, whereas *P. agglomerans* primarily promoted greater root surface area and leaf expansion. By contrast, *P. aryabhattai* induced only moderate growth responses, and the consortium treatment resulted in values comparable to or lower than those observed in the uninoculated control.

Under complete P deprivation (No P), bacterial inoculation significantly mitigated growth reduction in a strain-dependent manner (Table 1). Inoculation with *P. agglomerans* resulted in the highest shoot dry weight (SDW; 91%) and plant dry weight (PDW; 86%), as well as the greatest total leaf area (158%), compared with the uninoculated control. In contrast, *A. brasilense* exerted the strongest effects on root system architecture, significantly increasing root length by 23–56% and root surface area by 25–54% compared with all other treatments. The consortium treatment promoted biomass accumulation to a greater extent than *P. aryabhattai* but showed comparatively weaker effects on root system development and did not outperform the most effective single strains for either biomass or root-related parameters.

When insoluble P was supplied as the sole P source (No P + IP), the consortium treatment resulted in the highest root and shoot biomass, exceeding both the uninoculated control and all single-strain inoculations by more than 30%. In contrast, individual strains showed stronger effects on root system architecture. *P. agglomerans* promoted the greatest root length (46%) and root surface area (11%), whereas *A. brasilense* significantly increased root root volume by 47% relative to the control. *P. aryabhattai* induced moderate improvements in biomass and root traits. Overall, under No P + IP conditions, the consortium was most effective in enhancing plant biomass, while single-strain inoculations preferentially stimulated root system development.

SPAD values were primarily influenced by P supply, with higher values observed under P-limited conditions compared with treatments receiving soluble P. The effect of bacterial inoculation on SPAD was secondary and variable, showing strain-dependent modulation depending on the P level.

### Phosphorus accumulation in roots and leaves

**Fig. 2:**
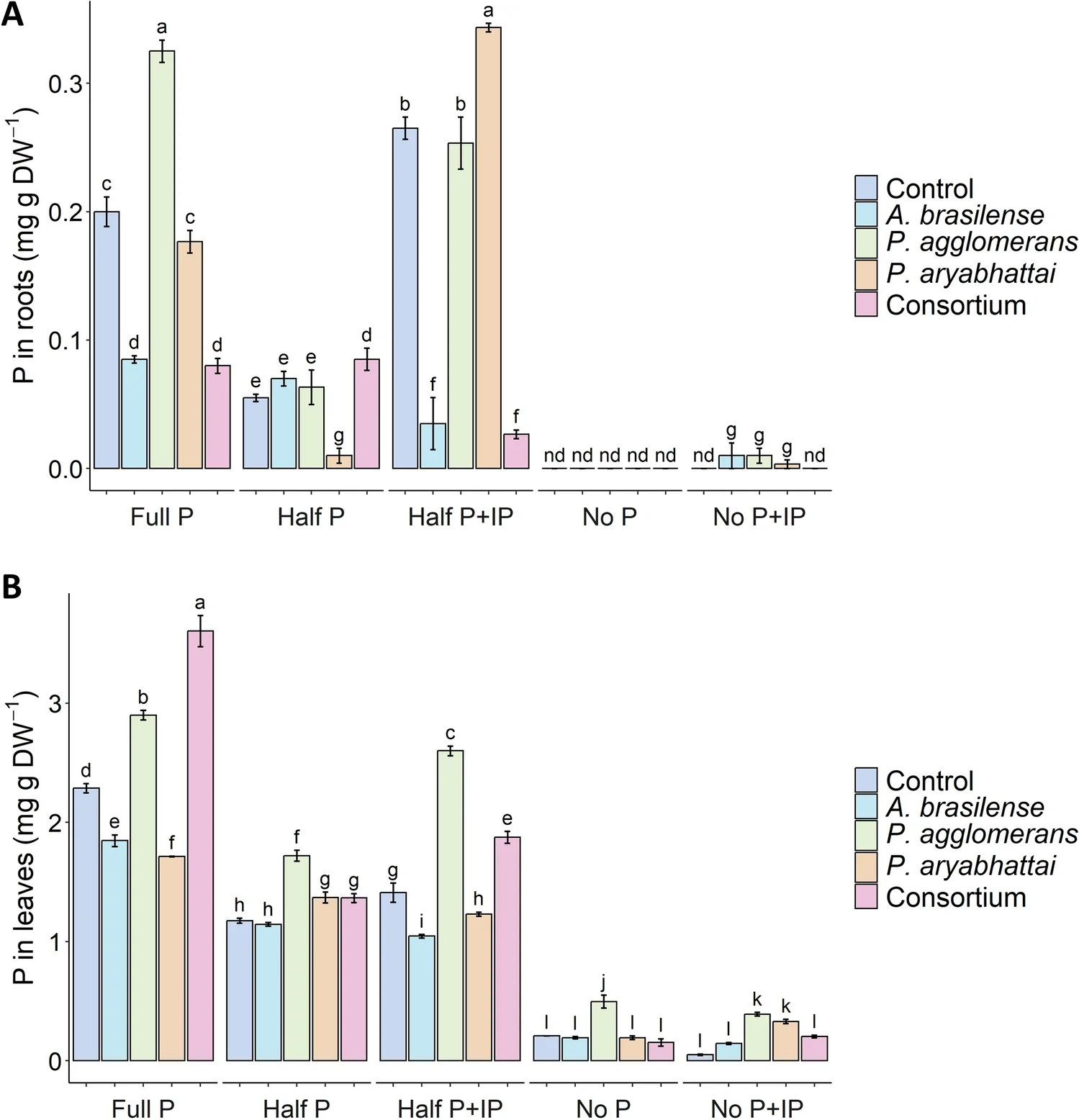
Total P content in the roots (**A**) and leaves (**B**) of tomato plants grown under five P levels and five bacterial treatments. Data are expressed as means ± SE (*n* = 3). Equal letters correspond to average values that do not differ significantly according to the Scott-Knott test (*P* < 0.05). nd, not detected

Phosphorus concentration in roots and leaves was strongly influenced by P supply and bacterial inoculation (BI), with a highly significant P × BI interaction (two-way ANOVA, *P* ≤ 0.001; Fig. 2, Suppl. Table S2), indicating that the effects of bacterial treatments depended on the P level. Under Full P conditions, inoculation with *P. agglomerans* resulted in a 65% increase in root P concentration relative to the uninoculated control. Conversely, root P levels were markedly lower following inoculation with *A. brasilense* and the consortium, declining by around 55% and 60%, respectively, whereas plants treated with *P. aryabhattai* showed values comparable to the control. In leaves, the consortium induced the strongest increase in P concentration (58%), followed by *P. agglomerans* (27%), whereas *A. brasilense* and *P. aryabhattai* were associated with reduced leaf P levels (19% and 25%, respectively).

As P availability decreased to Half P, P concentrations in both roots and leaves were markedly lower than under Full P conditions, with pronounced strain-dependent responses. In roots, inoculation with the consortium showed the strongest increase (54%), followed by *A. brasilense* (27%) and *P. agglomerans* (14%), whereas *P. aryabhattai* showed a pronounced reduction (82%) compared to the control. In leaves, *P. agglomerans* exhibited the highest P concentration (46%) relative to the control, while *P. aryabhattai* and the consortium showed only minor changes, remaining within ~16% of the uninoculated treatment.

Under combined soluble and insoluble P supply (Half P + IP), *P. aryabhattai* showed the highest root P concentration, increasing by approximately 29% relative to the control, while *P. agglomerans* exhibited values comparable to the control. In contrast, inoculation with *A. brasilense* and the consortium resulted in pronounced reductions in root P concentration by 87% and 90%, respectively. In leaves, *P. agglomerans* again showed the strongest response, increasing P concentration by approximately 84%, followed by the consortium (33%), whereas *A. brasilense* and *P. aryabhattai* exhibited lower values than the control.

In the absence of soluble P (No P), root P concentrations were below the detection limit for all treatments. Leaf P concentrations were uniformly low and comparable among treatments, with the exception of *P. agglomerans*, which showed a substantial increase (138%) relative to the control. When P was supplied exclusively in insoluble form (No P + IP), root P concentrations remained very low across all treatments, with values at or close to the detection limit. Leaf P concentrations, however, increased following inoculation with *P. agglomerans* and *P. aryabhattai*, whereas *A. brasilense* and the consortium showed values comparable to the uninoculated control.

### Network analysis of nutrient correlations in roots and leaves

**Fig. 3:**
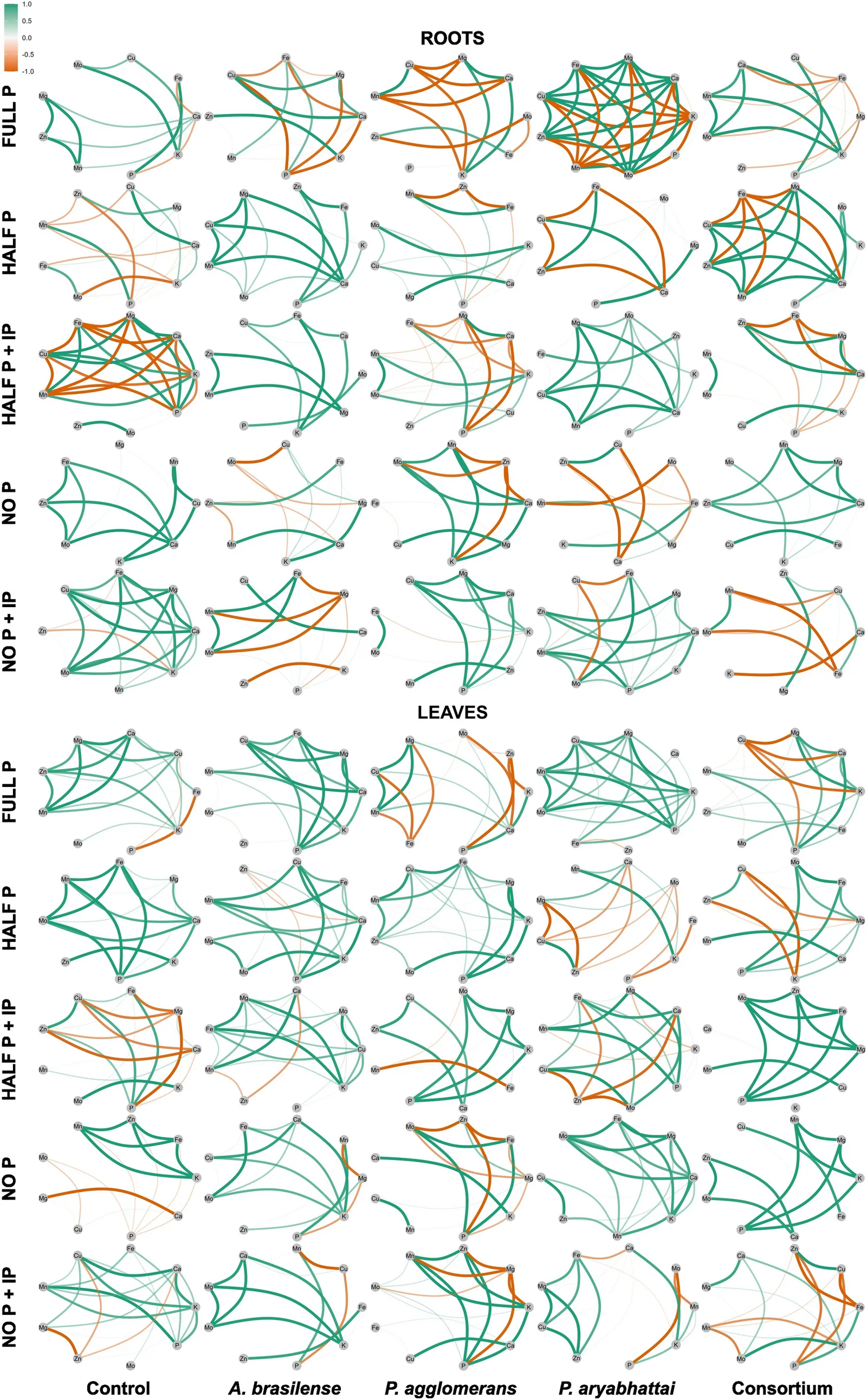
Ionomic correlation networks in tomato tissues across treatments. Grey nodes represent individual nutrients, and edges connect nutrients that are significantly correlated. Edges were defined by Pearson correlation coefficients with an absolute value ≥ 0.7, indicating strong positive or negative associations between nutrients. Edge color indicates the direction of the correlation (green, positive; orange, negative), while edge thickness reflects the absolute correlation coefficient (|r|). Networks are shown separately for roots and leaves across P supply conditions and bacterial treatments

Network density (ND) analysis showed pronounced tissue- and treatment-dependent differences in nutrient correlation structure (Fig. 3; Suppl. Table S3). Both network connectivity and the identity of highly connected nutrients varied across P levels and bacterial inoculation treatments. In roots under Full P conditions, *P. aryabhattai* exhibited the highest connectivity, characterized by a predominance of negative correlations (68%), indicating a highly structured but antagonistic nutrient network. By contrast, *A. brasilense*, *P. agglomerans*, and the consortium showed a more balanced distribution of positive and negative correlations (Fig. 3; Suppl. Table S3). Across treatments, Ca and Mg consistently acted as central nodes, frequently linked with K and selected micronutrients (Fig. 3). As P availability declined (Half P), overall connectivity remained comparable to the control in most inoculated treatments, although *P. aryabhattai* again exhibited the highest ND (62%). When soluble and insoluble P were supplied together (Half P + IP), the uninoculated control showed the greatest connectivity (67%), whereas inoculated treatments generally displayed intermediate or reduced density. Across these P-limited regimes, micronutrients including Fe, Mn, and Zn became more integrated within the networks, indicating a stronger coupling between macro- and micronutrient pools. Notably, the proportion of negative correlations increased in the uninoculated control under P limitation, whereas bacterial inoculation tended to moderate this shift. Under complete P deprivation (No P), P was not detected in roots and therefore did not participate as a network node. Connectivity was generally lower (39–46% density), and some treatments, including the control and consortium, exhibited exclusively positive correlations. When insoluble P was supplied alone (No P + IP), connectivity varied widely (36–75%), but Ca and Mg remained the main structural hubs across treatments (Fig. 3; Suppl. Table S3).

Leaf nutrient networks exhibited comparatively stable architecture across P regimes, with ND ranging from 31 to 61% and a general predominance of positive correlations (Fig. 3; Supplementary Table S3). Under Full P conditions, the highest connectivity was observed in *P. aryabhattai* (61% ND; 96% positive correlations), reflecting strong and largely coordinated nutrient associations. In contrast, *P. agglomerans* displayed a more balanced distribution of positive and negative correlations, indicating greater heterogeneity in nutrient interactions. With decreasing P availability (Half P), the highest density was recorded in *P. agglomerans* (58%), with exclusively positive correlations (100%), and the network was primarily organized around Fe, Cu, and Zn. Conversely, *P. aryabhattai* showed a high proportion of negative correlations (60%), largely involving interactions with Zn and Mo. When soluble and insoluble P were combined (Half P + IP), *A. brasilense* exhibited the most connected network (ND=58%) with predominantly positive associations (91%). In this case, strong links among Mg, K, Cu, and Fe defined the network core, while P itself showed weaker and more peripheral connections. Under severe P limitation (No P and No P + IP), leaf networks remained relatively structured but underwent clear reorganization. In the absence of soluble P (No P), *P. aryabhattai* and the consortium displayed exclusively positive correlations, mainly centered on Mn, Mg, K, and Ca. However, when P was supplied only in insoluble form (No P + IP), the same treatments exhibited a higher proportion of negative correlations (Fig. 3; Suppl. Table S3).

### Expression of P transporter-related genes

In tomato, seven genes involved in phosphate uptake belonging to the Phosphate Transporter Family 1 (PHT1) have been identified (*SlPHT1.1*-*SlPHT1.7*; Chen et al. 2014). However, under the present experimental conditions, the expression of *SlPHT1.2*, *SlPHT1.3*, *SlPHT1.4*, and *SlPHT1.5* was not detectable under P starvation (data not shown). Therefore, the relative expression of *SlPHT1.1*, *SlPHT1.6*, and *SlPHT1.7* was analyzed (Table 2), using the uninoculated Full P treatment as the reference (expression ~1). Relative to this baseline, each gene displayed a distinct transcriptional response to changes in P availability.

**Table 2:** Relative expression of *SlPHT1.1*, *SlPHT1.6*, and *SlPHT1.7* in tomato roots grown under different phosphorus supply levels (*P*) and bacterial inoculation treatments (BI)

Main factors	PHT1 transporter genes		
	***SlPHT1.1***	***SlPHT1.6***	***SlPHT1.7***
Full P x Control	1.18 ± 0.18 e	0.91 ± 0.09 f	0.87 ± 0.13 i
Full P x *A. brasilense*	0.39 ± 0.09 g	0.28 ± 0.07 g	0.28 ± 0.08 i
Full P x *P. agglomerans*	0.64 ± 0.04 f	0.60 ± 0.03 g	1.43 ± 0.11 i
Full P x *P. aryabhattai*	0.14 ± 0.03 g	1.51 ± 0.25 e	0.63 ± 0.07 i
Full P x Consortium	0.18 ± 0.01 g	0.25 ± 0.04 g	0.42 ± 0.01 i
Half P x Control	1.33 ± 0.22 e	1.38 ± 0.11 e	2.66 ± 0.06 i
Half P x *A. brasilense*	0.18 ± 0.01 g	4.13 ± 0.06 b	7.27 ± 0.21 i
Half P x *P. agglomerans*	0.62 ± 0.27 f	0.45 ± 0.05 g	2.38 ± 0.17 i
Half P x *P. aryabhattai*	0.70 ± 0.14 f	1.30 ± 0.22 e	4.84 ± 0.08 i
Half P x Consortium	1.16 ± 0.13 e	1.28 ± 0.13 e	5.98 ± 0.60 i
Half P+IP x Control	1.41 ± 0.03 e	5.61 ± 0.20 a	7.39 ± 0.56 i
Half P+IP x *A. brasilense*	2.25 ± 0.16 b	4.34 ± 0.23 b	21.15 ± 1.12 i
Half P+IP x *P. agglomerans*	2.57 ± 0.16 a	3.54 ± 0.30 c	1.67 ± 0.19 i
Half P+IP x *P. aryabhattai*	1.66 ± 0.24 c	2.67 ± 0.36 d	8.01 ± 0.60 i
Half P+IP x Consortium	0.23 ± 0.01 g	1.65 ± 0.40 e	11.39 ± 0.26 i
No P x Control	1.48 ± 0.11 d	3.40 ± 0.39 c	143.18 ± 6.27 e
No P x *A. brasilense*	0.31 ± 0.06 g	2.59 ± 0.17 d	186.81 ± 4.97 c
No P x *P. agglomerans*	2.74 ± 0.21 a	3.36 ± 0.33 c	330.63 ± 26.73 a
No P x *P. aryabhattai*	1.74 ± 0.16 c	2.83 ± 0.12 d	166.43 ± 0.63 d
No P x Consortium	1.18 ± 0.07 e	4.42 ± 0.34 b	247.16 ± 14.37 b
No P+IP x Control	1.27 ± 0.07 e	3.67 ± 0.28 c	156.45 ± 14.81 d
No P+IP x *A. brasilense*	0.21 ± 0.01 g	0.54 ± 0.04 g	161.98 ± 1.61 d
No P+IP x *P. agglomerans*	0.57 ± 0.06 f	1.65 ± 0.20 e	67.87 ± 1.40 g
No P+IP x *P. aryabhattai*	0.50 ± 0.07 f	1.27 ± 0.19 e	50.94 ± 0.13 h
No P+IP x Consortium	0.81 ± 0.16 f	3.38 ± 0.23 c	111.42 ± 5.52 f
Significance			
P level (*P*)	***	***	***
Bacterial inoculant (BI)	***	***	***
P x BI	***	***	***

All data are expressed as mean ± SE, *n* = 3

Different letters within each column indicate significant differences according to Scott–Knott test. The expression level of each gene was normalized to the expression level of the elongation factor isoform 1-alpha (*SlEF-1α*). The relative expression ratios were calculated using the uninoculated control under Full P as a reference sample

Two-way ANOVA followed by Scott–Knott test (*P* = 0.05) was applied for each growth parameter

^***^ denotes significance at *P* ≤ 0.001

For *SlPHT1.1*, transcript levels under Full P conditions were highest in uninoculated plants, whereas all inoculation treatments caused strong downregulation, with reductions ranging from approximately twofold to eightfold, particularly following *A. brasilense*, *P. aryabhattai*, and consortium inoculation. At Half P, *SlPHT1.1* expression increased slightly in the control relative to Full P, while inoculation effects became strain-dependent. The consortium maintained expression levels close to the control, whereas *A. brasilense* showed pronounced repression (~ sevenfold). When soluble and insoluble P were supplied together (Half P + IP), *SlPHT1.1* was induced by *A. brasilense* and *P. agglomerans*, reaching approximately twofold higher expression than the Full P control, while the consortium again exhibited strong downregulation. Under P deprivation (No P), *SlPHT1.1* expression remained elevated in the control and was further induced by *P. agglomerans* (> twofold), whereas *A. brasilense* consistently showed low expression across P-limited conditions. Under No P + IP, *SlPHT1.1* transcript levels declined in all treatments, with the lowest expression observed in *A. Brasilense-*inoculated plants.

In contrast to *SlPHT1.1*, *SlPHT1.6* displayed a more pronounced induction in response to decreasing P availability. Under Full P conditions, *SlPHT1.6* expression was downregulated in all inoculated plants except *P. aryabhattai*, which showed a moderate induction (~ 1.7-fold), while *A. brasilense* and the consortium exhibited the strongest repression (~ 3–4-fold). At Half P, *SlPHT1.6* expression increased markedly, with *A. brasilense* showing the highest induction (~ 4.5-fold relative to the Full P reference), whereas the consortium and *P. aryabhattai* maintained intermediate expression and *P. agglomerans* remained lower. A similar pattern persisted under Half P + IP, where *SlPHT1.6* expression remained elevated in the control and *A. brasilense* treatments (~ fourfold–sixfold), with intermediate levels in the other inoculated plants. Under P deprivation (No P), *SlPHT1.6* transcript levels were consistently high across treatments, reaching maximal values in the consortium (~ fivefold). When P was supplied exclusively in insoluble form (No P + IP), *SlPHT1.6* expression declined in *A. Brasilense*-inoculated plants but remained moderately elevated in the control and consortium treatments.

Among the analyzed transporters, *SlPHT1.7* showed the strongest transcriptional responsiveness to P availability. Under Full P conditions, *SlPHT1.7* expression was generally low, with only *P. agglomerans* displaying a modest induction (~ 1.6-fold), while all other inoculation treatments resulted in downregulation relative to the control. As P availability decreased to Half P, *SlPHT1.7* expression increased markedly in all treatments, with the strongest induction observed following *A. brasilense* (~ eightfold) and consortium inoculation (~ sevenfold). When soluble and insoluble P were supplied together (Half P + IP), *SlPHT1.7* transcript levels were further enhanced, reaching maximal induction in *A. brasilense*-inoculated plants (~ 24-fold), while *P. aryabhattai* and the consortium also maintained high expression (~ 9- and 13-fold, respectively). Under P deprivation (No P), *SlPHT1.7* expression increased drastically across all treatments, with the highest transcript levels detected in *P. agglomerans* and consortium-inoculated plants (~ 380- and 285-fold, respectively), and strong upregulation also observed in *A. brasilense* and *P. aryabhattai* (> 190-fold). In contrast, when P was supplied exclusively in insoluble form (No P + IP), *P. agglomerans* and *P. aryabhattai* showed a marked reduction in *SlPHT1.7* expression (57% and 67%, respectively), compared to the uninoculated control.

### Integrated clustering analysis of morpho-physiological, ionomic, and transcriptional traits

**Fig. 4:**
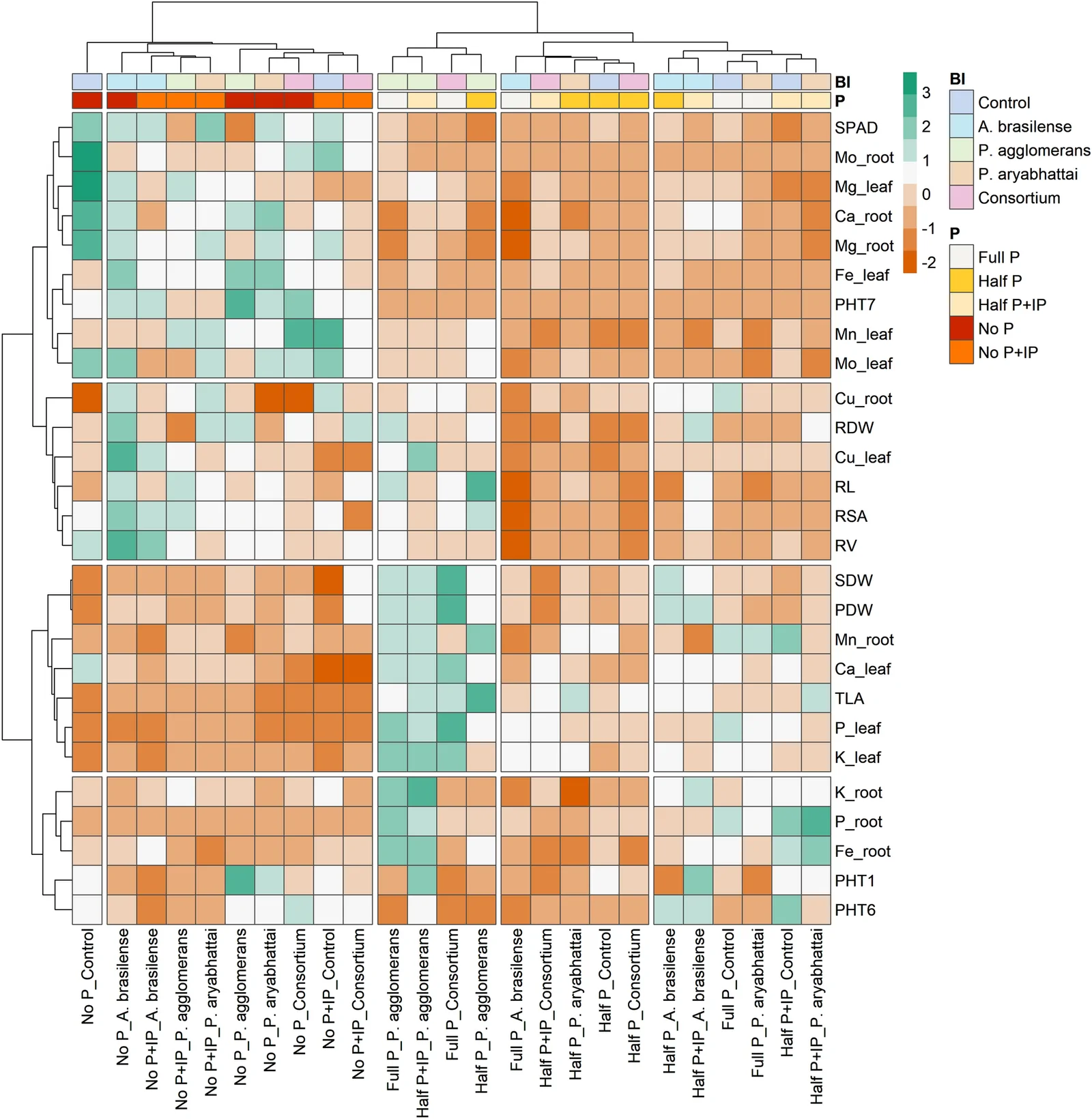
Heatmap cluster analysis summarizing the morpho-physiological, ionomic, and transcriptional responses of tomato plants across P levels (P) and bacterial inoculant (BI) treatments. Data were standardized per variable using *z*-scores. Color gradients represent relative values across treatments, with green indicating higher relative values, orange indicating lower relative values, and intermediate tones corresponding to values close to the mean for each parameter. *RDW* root dry weight, *SDW* shoot dry weight, *PDW* plant dry weight, *TLA* total leaf area, *RL* root length, *RSA* root surface area, *RV* root volume

Hierarchical clustering of all measured variables revealed a strong separation of treatments according to P availability (Fig. 4). Treatments lacking soluble P (No P and No P + IP) formed a distinct cluster, clearly separated from those receiving soluble P (Full P, Half P, and Half P + IP). This separation was associated with coordinated reductions in biomass-related traits, lower tissue P concentrations, and strong induction of phosphate transporter genes, particularly *PHT7*. Although treatments were grouped primarily according to P level, the uninoculated No P control formed a partially distinct branch within this cluster, indicating that bacterial inoculation induced slight measurable adjustments even under severe P limitation.

In contrast, when soluble P was available (Full P, Half P, and Half P + IP), clustering patterns reflected not only P supply but also bacterial inoculation. Within this broader group, treatments were further resolved into three subclusters shaped by strain-dependent effects (Fig. 4). The first subcluster (located on the left) grouped mainly *P. agglomerans* treatments across different soluble P levels, indicating a stable and consistent plant response associated with this strain. This cluster was characterized by comparatively higher shoot and root growth parameters and improved leaf nutrient status, suggesting that *P. agglomerans* promoted a stable growth and ionomic configuration under soluble P conditions, partially buffering moderate variations in P supply. The second subcluster (positioned in the middle) comprised treatments with an intermediate phenotypic profile, showing moderate growth stimulation and less pronounced modulation of nutrient-related traits compared with the *P. agglomerans* group. The third subcluster included *A. brasilense* treatments under reduced soluble P (Half P and Half P + IP), which were associated with enhanced aerial development relative to other treatments at comparable P levels. Notably, these samples clustered together with Full P_Control and Full P_*P. aryabhattai*, indicating that *A. brasilense* under moderate P supply induced a phenotypic and ionomic configuration resembling that observed under optimal P conditions.

## Discussion

Results here presented show that P availability is the primary driver of tomato growth responses, strongly shaping biomass allocation, root system architecture, and leaf physiological traits, thus confirming its central role in defining the plant response framework. Moreover, the highly significant interaction between P level and bacterial inoculation indicates that the effectiveness of PGPB is closely linked to the nutrient context, and not to an intrinsic property of the inoculant alone (Fernandes et al. 2025). This pattern is consistent with previous studies showing that the benefits of microbial associations tend to be strongest when nutrient availability is scarce, pushing plants to rely more strongly adaptive uptake mechanisms (Rosa et al. 2020; Consentino et al. 2022). Therefore, P availability appears to define the physiological background against which bacterial effects are expressed.

Across treatments, individual bacterial strains frequently outperformed the consortium in stimulating specific morphological traits, particularly those related to root system development, indicating that strain identity played a major role in determining plant responses. For example, *P. agglomerans* consistently promoted root elongation and surface expansion, traits known to enhance P acquisition efficiency in low-mobility soils (Luziatelli et al. 2020b). Such responses are commonly associated with bacterial modulation of root developmental pathways, including auxin-mediated stimulation of lateral roots and elongation zones (Luziatelli et al. 2020a, b). Similarly, *A. brasilense* showed a strong capacity to enhance root architectural traits under P-limited conditions, in line with its well-documented role in modifying root growth dynamics and nutrient uptake efficiency through phytohormone-related mechanisms (Pelagio-Flores et al. 2025). In contrast, the bacterial consortium did not consistently outperform single strains in promoting root system architecture, highlighting that the combination of multiple PGPB does not necessarily result in additive or synergistic effects under all nutritional conditions. Competition for root exudates, functional redundancy, or suboptimal strain interactions may limit the expression of individual traits within consortia, particularly under moderate nutrient limitation (Timofeeva et al. 2023; Samonty et al. 2025). In addition, the combined activity of multiple strains producing similar bioactive compounds, such as indole−3-acetic acid (IAA), may lead to non-linear or unbalanced effects at the root surface, potentially disrupting normal root development rather than enhancing it (Etesami and Glick 2024; Roca et al. 2026). These complex and not always positive responses may reflect differences in bacterial functional traits (e.g., auxin levels) and their interactions within the consortium, highlighting that consortium effectiveness depends on strain combination rather than on the mere number of strains included. Nevertheless, the consortium showed a clear advantage under the most restrictive conditions, particularly when insoluble P was the sole P source, leading to higher overall plant biomass. This observation is consistent with the hypothesis that microbial consortia may become especially valuable in complex or nutritionally harsh environments, possibly because multiple complementary mechanisms can act simultaneously to support plant nutrient use (Benmrid et al. 2025).

Consistent with these growth responses, bacterial inoculation also modulated P accumulation patterns in roots and leaves, indicating that structural changes in roots were accompanied by shifts in nutrient uptake and internal distribution. Because the experiment was conducted in hydroponics, where nutrient diffusion constraints and soil buffering effects are respectively negligible or even absent (Tuxun et al. 2025), differences in tissue P concentration cannot be solely attributed to the classical rhizosphere solubilization process. In this context, the strain-dependent differences in tissue P concentration observed here are consistent with the possibility that the tested bacteria influence P nutrition through distinct physiological and rhizochemical mechanisms (Stegelmeier et al. 2022). Across P levels, *P. agglomerans* showed the most consistent enhancement of P accumulation in the tissue, particularly by increasing P concentration in leaves under both adequate and limiting supply, suggesting a relatively stable effect on plant P nutrition. Such effects may reflect mechanisms previously described for *Pantoea* spp., including rhizosphere acidification, organic acid production, and modulation of membrane transport processes (Ma et al. 2023; Tian et al. 2024). In contrast, *A. brasilense* frequently reduced tissue P concentrations despite stimulating root growth. This apparent decoupling between biomass production and P accumulation suggests a dilution effect, where increased growth is not accompanied by proportional increases in P uptake (Schillaci et al. 2021). Such responses are consistent with reports that *Azospirillum* spp. primarily modulate root architecture and hormonal signaling (Cassán et al. 2014; Pelagio-Flores et al. 2025), potentially enhancing P use efficiency rather than total P acquisition. In addition, the persistence of very low root P concentrations under severe P deprivation confirms the strength of the imposed limitation in the hydroponic system. Nevertheless, the observed increases in leaf P following specific inoculations indicate that microbial treatments can influence internal P redistribution even when external P supply is constrained (Rosa et al. 2020).

Extending the analysis beyond absolute P concentrations, network evaluation revealed that P levels and bacterial inoculation reshaped the coordination among nutrients in distinct ways between root and leaf tissues. One of the most consistent patterns observed was the central role of Ca and Mg in root networks, regardless of P supply. These elements are essential for membrane stability, charge balance, and cellular signaling, and they strongly influence the uptake and movement of other cations (Tang and Luan 2017). Their recurring presence as central nodes in the network suggests that maintaining divalent cation balance provides a structural backbone for root ionomic organization (Baxter 2009). The shifts observed around these nodes in response to different bacterial strains may reflect changes in transporter activity or membrane dynamics induced by microbial signals. However, strains differed in how they reorganized the surrounding network. For instance, under Full P conditions, plants inoculated with *A. brasilense*, *P. agglomerans*, or the consortium displayed a more even distribution of positive and negative correlations, indicating a more coordinated ionomic structure, which may be consistent with microbial modulation of nutrient transport processes (Zuluaga et al. 2026b, a). Nonetheless, under complete P deprivation, the consortium did not consistently enhance network density compared with single strains, suggesting that a more coordinated ionomic structure under adequate P does not necessarily predict greater structural integration when P becomes limiting. Such context dependency aligns with observations from synthetic microbial communities, where combined strains can produce host responses that differ qualitatively but not always quantitatively from those induced by individual strains (Herrera Paredes et al. 2018). This root-level plasticity contrasts with the behavior observed in leaf tissues. In fact, leaf nutrient networks remained more stable and were largely dominated by positive correlations across P regimes, likely reflecting tighter systemic control of nutrient allocation in photosynthetic tissues, where coordinated mineral balance is essential for metabolic performance (Lu et al. 2025). Even under severe P limitation, the preservation of nutrient correlations in leaves suggests that internal redistribution mechanisms buffer external fluctuations, maintaining functional integration despite changes occurring at the root level. This difference between roots and leaves points to a stronger local plasticity belowground and a more buffered regulation in aboveground tissues.

Examining the expression patterns of the *PHT1* family at the root level showed that P availability was the primary determinant of the transcriptional responses. The strong induction of *SlPHT1.6* and especially *SlPHT1.7* under decreasing P supply is consistent with the established role of high-affinity *PHT1* transporters in P acquisition during P starvation (Nussaume 2011), highlighting their central contribution to P uptake and adaptive responses in tomato under P-limiting conditions. In contrast, *SlPHT1.1* showed weaker or even suppressed expression in several treatments, indicating functional differentiation within the PHT1 family. Such specialization has been described in tomato and other crops, where individual transporters differ in regulatory sensitivity and physiological contribution (Chen et al. 2014). In addition to P availability, the impact of bacterial inoculation on *PHT1* expression was strain-dependent and did not consistently enhance transporter induction. Under P limitation, *A. brasilense* and *P. agglomerans* appeared to enhance the intrinsic P-starvation signaling response of the plant, consistent with evidence that beneficial rhizobacteria can influence host nutrient transporter networks through hormonal and signaling interactions, in addition to direct nutrient mobilization (Saia et al. 2015). Conversely, the reduced expression of *PHT1* transporters by bacterial treatments when insoluble P was supplied suggests a partial attenuation of starvation signaling, possibly reflecting increases in internal P status that could be perceived through systemic sensing pathways coordinating shoot-to-root signaling and repress high-affinity transporter expression when P is perceived (Yang et al. 2024). Although indirect, this interpretation suggests that bacterial inoculation may influence not only P acquisition itself, but also the signaling networks that regulate plant perception of P status.

In the integrative clustering analysis, traits assembled into coherent response profiles, indicating that tomato plants reorganize multiple functional layers simultaneously in response to nutrient availability and microbial interaction. Under conditions of limited soluble P, the clustering pattern suggests a distinct adaptive configuration characterized by transcriptional activation of P transporters alongside constrained biomass development, suggesting that the plant reallocates its resources toward enhancing nutrient acquisition mechanisms instead of investing in growth (Paz-Ares et al. 2022). When soluble P was available, plants inoculated with *P. agglomerans* tended to maintain a consistent balance between growth performance and nutrient status, even across moderate variations in P supply, suggesting a more coordinated regulation of nutrient uptake and allocation within the plant (Saadouli et al. 2021). Notably, the close association between the consortium and *P. agglomerans* under Full P conditions indicates that, when P was not limiting, the consortium elicited a plant response closely resembling that induced by *P. agglomerans*, suggesting that this strain may have exerted a relatively stronger influence on the consortium response. In fact, previous studies have shown that specific members of a consortium can be selectively activated by rhizosphere signals and host ecophysiological responses to express their growth-promoting effects, implying differential contributions of individual strains in multi-strain inoculants (Bradáčová et al. 2019). Finally, the clustering analysis shows that microbial effects were not restricted to individual traits, but involved coordinated changes in transporter expression, nutrient accumulation, and growth parameters, forming integrated response patterns. Several studies have reported that beneficial microbes shape plant performance by adjusting overall regulatory balance and nutrient management strategies, contributing to greater nutritional flexibility instead of simply stimulating one specific physiological process (Zuluaga et al. 2023, 2024; Liu et al. 2025). Overall, the present results support the view that the effects of PGPB on tomato are strongly context-dependent and emerge from the interaction among P availability, strain identity, and plant regulatory responses. The strain-specific responses observed throughout this study clearly demonstrate that bacterial inoculation influenced plant performance under the different P regimes. However, because bacterial persistence and root colonization were not directly assessed following the initial inoculation, the mechanisms underlying these responses cannot be fully resolved. Consequently, although all treatments were subjected to the same nutrient solution renewal regime, it was not possible to distinguish the relative contribution of sustained bacterial colonization from that of transient early plant–microbe interactions. Future studies combining colonization analyses with plant phenotyping will help clarify how bacterial persistence shapes the performance of individual strains and bacterial consortia under contrasting P availability.

## Conclusion

By integrating morpho-physiological, ionomic, and transcriptional responses across contrasting P availability and forms, this work showed that P availability emerged as the main factor shaping plant responses, while bacterial strains introduced strain-specific modulation of growth and nutrient traits. This integrative approach revealed that individual strains frequently induced targeted adjustments in root architecture, tissue P accumulation, and phosphate transporter expression, thereby highlighting a clear functional specialization of microbial effects. In particular, *P. agglomerans* showed stable and consistent performance under soluble P conditions, while *A. brasilense* was particularly effective in stimulating adaptive responses under moderate P limitation. Although *P. aryabhattai* generally induced more moderate growth responses, it contributed to distinct ionomic reorganization patterns and showed responsiveness under insoluble P supply, indicating a specific regulatory influence on nutrient networks. The consortium performed comparably to *P. agglomerans* under Full P conditions and showed advantages when insoluble P was the sole source, but it did not consistently outperform single strains across all scenarios. Taken together, these findings indicate that increasing inoculant complexity does not automatically translate into improved plant performance and that the relative effectiveness of single strains and consortia is strongly context-dependent. Combining strains with overlapping functional traits may fail to produce additive effects and can occasionally generate counterproductive responses. Moreover, it should be highlighted that, because this study focused on the early developmental phase of tomato, the observed responses capture processes occurring during root system establishment, a stage that is particularly sensitive to nutrient availability and microbial signaling. Therefore, the relative performance of single strains and consortia may also be strongly dependent on plant developmental stage, and further investigations at later growth phases are essential to assess the long-term stability and agronomic relevance of these microbial effects. Overall, improving P responses through microbial inoculation requires the strategic selection of compatible and complementary strains tailored not only to specific nutrient contexts but also to the plant developmental stage. In this sense, rational consortium design should prioritize functional balance and ecological compatibility, instead of the simple accumulation of plant growth-promoting traits or increased inoculant complexity.

## Supplementary Information

Below is the link to the electronic supplementary material.

Supplementary file1 (DOCX 38 KB)

## Data Availability

The data that support the findings of this study are available within the manuscript as regular data or supplementary information.

## Abbreviations

IP: Insoluble phosphorus
ND: Network density
P: Phosphorus
PGPB: Plant growth-promoting bacteria
PHT1: Phosphate transporter family 1
