# Supplementary material for: More is not always better: phosphorus context shapes the effectiveness of single-strain and consortium plant growth-promoting bacteria in tomato
Source: Planta. 2026 Aug 25;264(4):108. doi: 10.1007/s00425-026-05134-4 (PMC13506622; doi:10.1007/s00425-026-05134-4)
Supplement: Supplementary file 1 [file 425_2026_5134_MOESM1_ESM.docx]

**Supporting Information**

**More is not always better: phosphorus context shapes the effectiveness of single-strain and consortium PGPB in tomato**

Alice Ferreira Alves, Mónica Yorlady Alzate Zuluaga, Talita de Oliveira Caretta, Sonia Monterisi, Fernando Dini Andreote, Stefano Cesco, Youry Pii

**Table S1** Sequence of forward and reverse primers used in Real-time RT-PCR experiments.

| **Gene** | **Forward primer 5-3** | **Reverse primer 5-3** |
| --- | --- | --- |
| *SlPHT1.1* | GTTTTAGACAAGTTATCAGTTAG | ATTCCATCTTCTTATAACAAACC |
| *SlPHT1.2* | TGGAGAAGCATGAGATCATTAA | AACAAACAAGCTTACACAATACA |
| *SlPHT1.3* | TCATTTAAGATCGTGCAAAGAG | ATGATTATTTCATCGTAACATACA |
| *SlPHT1.4* | ATGAAGACTAGCAAACCTGTC | TGAAGATAGAAAGCACAAGGC |
| *SlPHT1.5* | GAGACAAAAGGCAGGTCACT | GCCAGAGTCAGTCCTATTAC |
| *SlPHT1.6* | TCAATGCACTTGGAATGTTATG | CTCCAGTTATAGTTTCTTGTG |
| *SlPHT1.7* | TGTTCCTGTGTAAAAGCAGTTA | CAACTATAAGAACATATGAAGGA |
| *SlEF1a* | ACTGTGCAGTGTGATGCTGG | AATACACAACTCAAAGACCAAAC |

**Table S2** Phosphorus concentration in roots and leaves (mg g⁻¹ DW) of tomato plants grown under different phosphorus supply levels (P) and bacterial inoculation treatments (BI).

| **Main factors** | **P in roots (mg g DW^-1^)** | **P in leaves (mg g DW^-1^)** |  |
| --- | --- | --- | --- |
|  |  |  |  |
| Full P x Control | 0.20 ± 0.02 c | 2.29 ± 0.07 d |  |
| Full P x *A. brasilense* | 0.09 ± 0.01 d | 1.85 ± 0.09 e |  |
| Full P x *P. agglomerans* | 0.33 ± 0.02 a | 2.90 ± 0.07 b |  |
| Full P x *P. aryabhattai* | 0.18 ± 0.02 c | 1.72 ± 0.01 f |  |
| Full P x Consortium | 0.08 ± 0.01 d | 3.61 ± 0.23 a |  |
| Half P x Control | 0.06 ± 0.01 e | 1.18 ± 0.04 h |  |
| Half P x *A. brasilense* | 0.07 ± 0.01 e | 1.15 ± 0.03 h |  |
| Half P x *P. agglomerans* | 0.06 ± 0.02 e | 1.72 ± 0.08 f |  |
| Half P x *P. aryabhattai* | 0.01 ± 0.00 g | 1.37 ± 0.08 g |  |
| Half P x Consortium | 0.09 ± 0.02 d | 1.37 ± 0.07 g |  |
| Half P+IP x Control | 0.27 ± 0.02 b | 1.41 ± 0.14 g |  |
| Half P+IP x *A. brasilense* | 0.04 ± 0.02 f | 1.05 ± 0.03 i |  |
| Half P+IP x *P. agglomerans* | 0.25 ± 0.04 b | 2.60 ± 0.07 c |  |
| Half P+IP x *P. aryabhattai* | 0.34 ± 0.01 a | 1.23 ± 0.03 h |  |
| Half P+IP x Consortium | 0.03 ± 0.01 f | 1.88 ± 0.09 e |  |
| No P x Control | nd | 0.21 ± 0.00 l |  |
| No P x *A. brasilense* | nd | 0.19 ± 0.02 l |  |
| No P x *P. agglomerans* | nd | 0.50 ± 0.10 j |  |
| No P x *P. aryabhattai* | nd | 0.19 ± 0.03 l |  |
| No P x Consortium | nd | 0.15 ± 0.06 l |  |
| No P+IP x Control | nd | 0.05 ± 0.01 l |  |
| No P+IP x *A. brasilense* | 0.01 ± 0.01 g | 0.15 ± 0.02 l |  |
| No P+IP x *P. agglomerans* | 0.01 ± 0.01 g | 0.39 ± 0.03 k |  |
| No P+IP x *P. aryabhattai* | 0.01 ± 0.00 g | 0.33 ± 0.03 k |  |
| No P+IP x Consortium | nd | 0.20 ± 0.02 l |  |
|  |  |  |  |
| Significance |  |  |  |
| P level (P) | *** | *** |  |
| Bacterial inoculant (BI) | *** | *** |  |
| P x BI | *** | *** |  |

All data are expressed as mean ± SE, *n* = 3. Two-way ANOVA followed by Scott-Knott test (*P* = 0.05) was applied for each growth parameter. *** denote significance at *P* ≤ 0.001. Different letters within each column indicate significant differences according to Scott-Knott test.

**Table S3** Network properties of the plant ionome across treatments.

| **Tissue** | **P level** | **Bacterial Inoculant** | **Nodes (nutrients)** | **Edges (correlations)** | **Network density ND (%)** | **Mean degree** | **Positive correlations (%)** | **Negative correlations (%)** |
| --- | --- | --- | --- | --- | --- | --- | --- | --- |
| Roots | Full P | Control | 9 | 13 | 36 | 3 | 85 | 15 |
|  |  | *A. brasilense* | 8 | 15 | 54 | 4 | 57 | 43 |
|  |  | *P. agglomerans* | 9 | 13 | 36 | 3 | 46 | 54 |
|  |  | *P. aryabhattai* | 9 | 36 | 100 | 8 | 32 | 68 |
|  |  | Consortium | 9 | 16 | 44 | 4 | 56 | 44 |
|  | Half P | Control | 9 | 18 | 50 | 4 | 39 | 61 |
|  |  | *A. brasilense* | 8 | 14 | 50 | 4 | 100 | 0 |
|  |  | *P. agglomerans* | 9 | 14 | 39 | 3 | 71 | 29 |
|  |  | *P. aryabhattai* | 7 | 13 | 62 | 4 | 54 | 46 |
|  |  | Consortium | 9 | 18 | 50 | 4 | 72 | 28 |
|  | Half P+IP | Control | 9 | 24 | 67 | 5 | 50 | 50 |
|  |  | *A. brasilense* | 9 | 11 | 31 | 2 | 100 | 0 |
|  |  | *P. agglomerans* | 9 | 20 | 56 | 4 | 45 | 55 |
|  |  | *P. aryabhattai* | 9 | 18 | 50 | 4 | 78 | 22 |
|  |  | Consortium | 9 | 15 | 42 | 3 | 60 | 40 |
|  | No P | Control | 8 | 11 | 39 | 3 | 100 | 0 |
|  |  | *A. brasilense* | 8 | 13 | 46 | 3 | 54 | 46 |
|  |  | *P. agglomerans* | 8 | 13 | 46 | 3 | 54 | 46 |
|  |  | *P. aryabhattai* | 8 | 12 | 43 | 3 | 33 | 67 |
|  |  | Consortium | 8 | 11 | 39 | 3 | 100 | 0 |
|  | No P+IP | Control | 8 | 21 | 75 | 5 | 86 | 14 |
|  |  | *A. brasilense* | 9 | 15 | 42 | 3 | 67 | 33 |
|  |  | *P. agglomerans* | 9 | 14 | 39 | 3 | 86 | 14 |
|  |  | *P. aryabhattai* | 9 | 22 | 61 | 5 | 86 | 14 |
|  |  | Consortium | 8 | 10 | 36 | 3 | 50 | 50 |
| Leaves | Full P | Control | 9 | 19 | 53 | 4.2 | 90 | 10 |
|  |  | *A. brasilense* | 9 | 18 | 50 | 4.0 | 94 | 6 |
|  |  | *P. agglomerans* | 9 | 17 | 47 | 3.8 | 47 | 53 |
|  |  | *P. aryabhattai* | 9 | 22 | 61 | 4.9 | 96 | 4 |
|  |  | Consortium | 9 | 20 | 56 | 4.4 | 65 | 35 |
|  | Half P | Control | 9 | 16 | 44 | 3.6 | 100 | 0 |
|  |  | *A. brasilense* | 9 | 18 | 50 | 4.0 | 83 | 17 |
|  |  | *P. agglomerans* | 9 | 21 | 58 | 4.7 | 100 | 0 |
|  |  | *P. aryabhattai* | 9 | 15 | 42 | 3.3 | 40 | 60 |
|  |  | Consortium | 9 | 15 | 42 | 3.3 | 60 | 40 |
|  | Half P+IP | Control | 9 | 16 | 44 | 3.6 | 66 | 34 |
|  |  | *A. brasilense* | 9 | 21 | 58 | 4.7 | 91 | 9 |
|  |  | *P. agglomerans* | 9 | 13 | 36 | 2.9 | 77 | 23 |
|  |  | *P. aryabhattai* | 9 | 16 | 44 | 3.6 | 72 | 28 |
|  |  | Consortium | 9 | 12 | 33 | 2.7 | 100 | 0 |
|  | No P | Control | 9 | 15 | 42 | 3.3 | 47 | 53 |
|  |  | *A. brasilense* | 9 | 15 | 42 | 3.3 | 80 | 20 |
|  |  | *P. agglomerans* | 9 | 14 | 39 | 3.1 | 57 | 43 |
|  |  | *P. aryabhattai* | 8 | 19 | 68 | 4.8 | 100 | 0 |
|  |  | Consortium | 9 | 11 | 31 | 2.4 | 100 | 0 |
|  | No P+IP | Control | 9 | 20 | 56 | 4.4 | 80 | 20 |
|  |  | *A. brasilense* | 9 | 13 | 36 | 2.9 | 77 | 23 |
|  |  | *P. agglomerans* | 9 | 18 | 50 | 4.0 | 72 | 28 |
|  |  | *P. aryabhattai* | 9 | 16 | 44 | 3.6 | 56 | 44 |
|  |  | Consortium | 9 | 17 | 47 | 3.8 | 53 | 47 |

Nodes correspond to nutrients, and edges represent significant pairwise correlations above a predefined correlation threshold (|r| ≥ 0.7). Network density (ND) was calculated as the ratio between the number of observed significant edges and the total number of possible edges. Mean degree was computed as twice the number of edges divided by the number of nodes. The percentages of positive and negative edges were calculated relative to the total number of significant correlations within each network.
